# Yellow Fever Vaccine—Reply to S. Arya

**DOI:** 10.3201/eid0503.990334

**Published:** 1999

**Authors:** T.P. Monath, J.A. Giesberg, E.G. Fierros

**Affiliations:** OraVax, Cambridge, Massachusetts, USA


tion centers in Lagos was fully potent, but potency in Osun and Oyo was 016 $\log_{10}$ to 0.22 $\log_{10}$ lower than the stipulated level (2). Furthermore, the titer of two vaccine lots that had been frozen after reconstitution from their lyophilized state dropped from the initial 3.15 $\log_{10}$ to 3.53 $\log_{10}$ to zero.

If the United States were to implement an extended strategy, similar studies of vaccine lots should be conducted to determine whether every vaccinee has received a full dose of yellow fever vaccine. In Illinois during the early 1970s, weak links in maintenance of refrigeration facilities and use of outdated vaccines in vials exposed to the sun for long hours were reported for live poliovirus vaccines (3). In the Northern Territory of Australia, examination of 144 vials of hepatitis B vaccine formulations during transport to immunization centers showed that 47.5% had been exposed to temperatures of -3°C or lower (4).

Assays of the potency of yellow fever vaccine, as well as quantification of vaccine-induced neutralizing antibody, is a multistep procedure that relies on inoculation of mice or Vero or polysaccharide cells (5). The successful "take" of yellow fever vaccine can be determined starting the second postvaccination day by demonstrable viremia detected by reverse-transcriptase polymerase chain reaction and by marked increases in neopterin, beta2-microglobulin, and circulating $CD8^+$ cells (6). Alternatively, elevated levels of tumor necrosis factor and interleukin-1 receptor antagonists on day two after vaccination (7) could be used to monitor the success of vaccinations by primary-care providers in remote areas in the United States (1) and elsewhere.

During the 1990s, isolation of yellow fever virus was reported in persons with a nonspecific febrile illness that did not meet the case definition of yellow fever (8). Air travel by such persons to the United States, which has areas infested by *Aedes aegypti,* could initiate yellow fever epidemics; because these travelers would have a nonspecific febrile illness, they would escape the existing surveillance network.

In conclusion, introducing yellow fever immunizations by primary health-care providers would be ideal, only with a concurrent plan to monitor vaccine potency at immunization centers and obtain in vitro evidence of a successful vaccine take. Such a strategy would blunt yellow fever–associated deaths, illnesses, and symptomless viral carriage in the community.

**Subhash C. Arya**
Centre for Logistical Research and Innovation
New Delhi, India

### References

1. Monath TP, Giesberg JA, Fierros EG. Does restricted distribution limit access and coverage of yellow fever vaccine in the United States? Emerg Infect Dis 1998:4:698-702.
2. Adu FD, Adedeji AA, Esan JS, Odusanya OG. Live viral vaccine potency: an index for assessing the cold chain system. Public Health 1996;110:325-30.
3. Rasmussen CM, Thomas CW, Mulrooney RJ, Morrissey RA. Inadequate poliovirus immunity levels in immunised Illinois children. Am J Dis Child 1973;126:465-9.
4. Miller NC, Harris MF. Are childhood immunization programmes in Australia at risk? Investigations of the cold chain in the Northern Territory. Bull WHO 1994;72:401-8.
5. World Health Organization. Techniques for potency evaluation of yellow fever vaccine. Technical Report Series 1998;872:67-8.
6. Reinhardt B, Jaspert R, Niedrig M, Kostner C, L'age-Stehr J. Development of viremia and humoral and cellular parameters of immune activation after vaccination with yellow fever virus strain 17D: a model of human flavivirus infection. J Med Virol 1998;56:159-67.
7. Hacker UT, Jelinek T, Erhardt S, Eigier A, Hartmann G, Nothdurft HD, et al. In vivo syntheses of tumor necrosis factor-alpha in healthy humans after live yellow fever vaccination. J Infect Dis 1998;177:774-8.
8. Sanders EJ, Maffin AA, Tukei PM, Kuria G, Ademba G, Agata NN, et al. First recorded outbreak of yellow fever in Kenya, 1992-1993. I. Epidemiologic investigations. Am J Trop Med Hyg 1998;59:644-9.

### Yellow Fever Vaccine—Reply to S. Arya

**To the Editor:** Dr. Arya correctly points out that there have been problems with degradation of live viral vaccines, including yellow fever vaccines, that have not been properly handled and stored at the point of use. However, in the United States and western Europe, yellow fever vaccines are stabilized and require the same storage facilities at the point of use as other vaccines routinely distributed by family physicians and pediatricians. Varicella vaccine (and even measles vaccine) is less stable than yellow fever vaccine but is distributed to all registered physicians in the United States. Since vaccines and other perishable medicines are typically shipped by overnight courier services using qualified methods that ensure maintenance of low temperature, there is no barrier to use of a similar system for yellow fever vaccine.

Empirical testing for antibody, viremia, or even surrogate markers of T-cell activation may be useful; however, it is difficult and expensive, involves unvalidated tests with unknown sensitivity and specificity, and is unnecessary, except under very special circumstances. A more direct measure of vaccine stability is direct potency measurement of samples stored at the point of use, as was done in the cited study in Nigeria by Adu et al. However, given the current controls on vaccine distribution in the United States, we do not believe that there would be a need to validate vaccine effectiveness at point of use in the event of a change of policy with respect to vaccinating centers. The cold-chain infrastructure and the training of medical personnel in vaccine storage and administration may not provide the same assurances in other countries. While our suggested changes to the system of yellow fever distribution may improve vaccine coverage and have other desirable benefits in the United States, they would not be appropriate for less stable systems for vaccine supply and use.

**T.P. Monath, J.A. Giesberg, and E.G. Fierros**
OraVax, Cambridge, Massachusetts, USA

**To the Editor:** Dr. Arya correctly points out that there have been problems with degradation of live viral vaccines, including yellow fever vaccines, that have not been properly handled and stored at the point of use. However, in the United States and western Europe, yellow fever vaccines are stabilized and require the same storage facilities at the point of use as other vaccines routinely distributed by family physicians and pediatricians. Varicella vaccine (and even measles vaccine) is less stable than yellow fever vaccine but is distributed to all registered physicians in the United States. Since vaccines and other perishable medicines are typically shipped by overnight courier services using qualified methods that ensure maintenance of low temperature, there is no barrier to use of a similar system for yellow fever vaccine.

Empirical testing for antibody, viremia, or even surrogate markers of T-cell activation may be useful; however, it is difficult and expensive, involves unvalidated tests with unknown sensitivity and specificity, and is unnecessary, except under very special circumstances. A more direct measure of vaccine stability is direct potency measurement of samples stored at the point of use, as was done in the cited study in Nigeria by Adu et al. However, given the current controls on vaccine distribution in the United States, we do not believe that there would be a need to validate vaccine effectiveness at point of use in the event of a change of policy with respect to vaccinating centers. The cold-chain infrastructure and the training of medical personnel in vaccine storage and administration may not provide the same assurances in other countries. While our suggested changes to the system of yellow fever distribution may improve vaccine coverage and have other desirable benefits in the United States, they would not be appropriate for less stable systems for vaccine supply and use.
